# A Practical Treatise on the Diseases of Infancy and Childhood

**Published:** 1859-10

**Authors:** 


ART. V.—*A Practical Treatise on the Diseases of Infancy and Childhood.* By T. H. TANNER, M. D., F. L. S., etc. Philadelphia : Lindsay & Blakiston, 1859. 12mo. pp. 464.

The objects and scope of this Treatise are expressed in the preface, with a conciseness which is in keeping with the remainder of the work. We quote the preface entire :

"The following pages have been written with the view of presenting to the student and the practitioner of medicine a complete work on the diseases of infants and children, within a moderate compass ; together with such a series of observations upon the hygienic and general management of the young as may lead to the prevention of much of the disease which now exists.

"In performing my task, my aim has been always to avoid too great diffuseness ; as well as to supply my readers with such facts as may lead them to think for themselves, rather than to overburden their memories with the various opinions of others."

The small size of the book, and the range of subjects which it embraces, render great brevity necessary. The author has made the best possible use of the space to which he has confined himself. As a hand-book, it is to be commended; while it does not supersede the necessity of the larger works of West, Condie, Meigs, and others. The practical views inculcated, so far as we have examined the work, seem to us to be in accordance with our present knowledge of pathology and therapeutics.

